# Design of an Always-On Image Sensor Using an Analog Lightweight Convolutional Neural Network

**DOI:** 10.3390/s20113101

**Published:** 2020-05-30

**Authors:** Jaihyuk Choi, Sungjae Lee, Youngdoo Son, Soo Youn Kim

**Affiliations:** 1 Department of Semiconductor Science, Dongguk University-Seoul, Seoul 04620, Korea; cjh9412@dongguk.edu; 2 Department of Industrial and Systems Engineering, Dongguk University-Seoul, Seoul 04620, Korea; sungjaelee@dgu.ac.kr

**Keywords:** always-on, Complementary Metal Oxide Semiconductor (CMOS) image sensor, convolutional neural networks, image classification

## Abstract

This paper presents an always-on Complementary Metal Oxide Semiconductor (CMOS) image sensor (CIS) using an analog convolutional neural network for image classification in mobile applications. To reduce the power consumption as well as the overall processing time, we propose analog convolution circuits for computing convolution, max-pooling, and correlated double sampling operations without operational transconductance amplifiers. In addition, we used the voltage-mode MAX circuit for max pooling in the analog domain. After the analog convolution processing, the image data were reduced by 99.58% and were converted to digital with a 4-bit single-slope analog-to-digital converter. After the conversion, images were classified by the fully connected processor, which is traditionally performed in the digital domain. The measurement results show that we achieved an 89.33% image classification accuracy. The prototype CIS was fabricated in a 0.11 μm 1-poly 4-metal CIS process with a standard 4T-active pixel sensor. The image resolution was 160 × 120, and the total power consumption of the proposed CIS was 1.12 mW with a 3.3 V supply voltage and a maximum frame rate of 120.

## 1. Introduction

**Figure 1:**
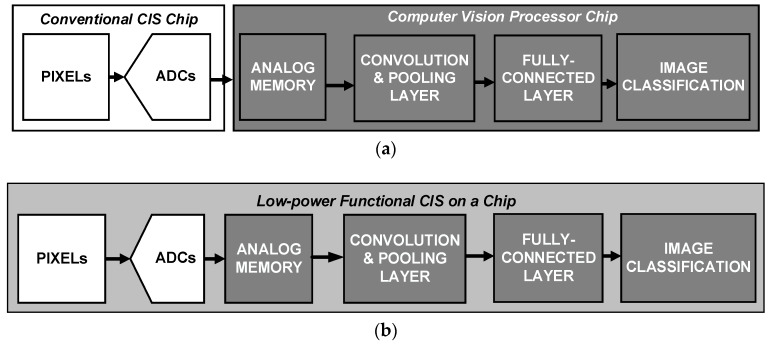
(**a**) Conventional image classification pipeline using a vision processor (two-chip system), and (**b**) the low-power face detection/recognition system in a chip.

**Figure 2:**
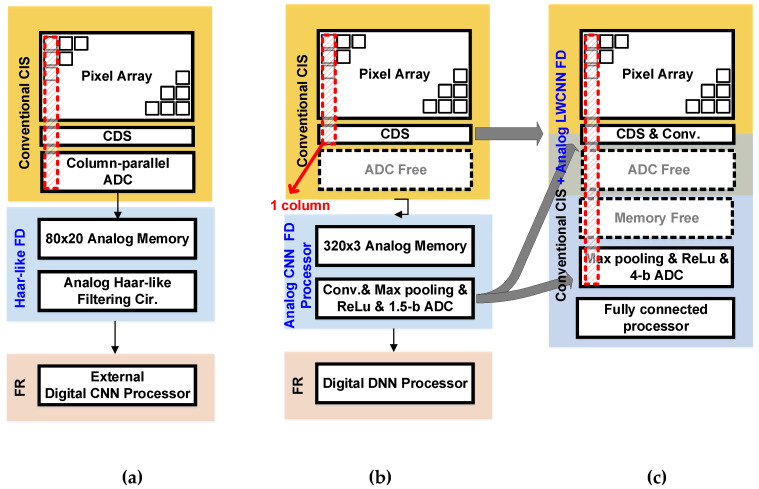
Different types of face detection (FD) system architectures for (**a**) Haar-like FD [12], (**b**) analog–digital hybrid CNN FD [13], and (**c**) the proposed analog lightweight CNN (a-LWCNN) FD.

In recent years, as the number of smart devices has increased with the rise of the Internet of Things [1], the importance of user authentication has increased as well. As an example of user-authentication applications, always-on face detection/recognition is highly convenient because direct physical contact, such as fingerprint scanning, is unnecessary [2,3,4]. However, integrating always-on face detection/recognition into mobile devices is challenging because of these devices’ limited battery life and thus limited power [5,6,7,8,9,10,11]. Therefore, a low-resolution and always-on Complementary Metal Oxide Semiconductor (CMOS) image sensor (CIS) that enables high power consuming devices, like ultra-high resolution (>tens of megapixels) CISs, to turn on for iris identification and face identification have received great attention [5]. Conventionally, classifying images for user authentication in mobile devices requires a conventional Complementary Metal Oxide Semiconductor image sensor (CIS) chip and a computer vision processor (CVP) chip, as shown in Figure 1a. In the CIS, the light intensity that accumulates in the pixel array is converted into the corresponding voltage, which is finally transmitted into the digital domain with column-parallel analog-to-digital converters (ADC). The pixel data are transferred to an external CVP chip and stored in analog memory blocks before a complex deep convolutional neural network (CNN) operation in the CVP that allows the classification of large datasets [12]. In this case, transmitting the data to another chip that is unsuitable for low-power operation requires a great deal of power [8,9,10,11]. In order to enhance the power efficiency, either the parts of the CNN circuits or that of the CVP can be implemented with the CIS in a chip, as shown in Figure 1b. In this case, since the CIS and CNN can be performed in a single chip, redundant and power-hungry blocks like ADC can be eliminated to further reduce power consumption [13]. Figure 2a,b shows the different types of low-power face detection (FD) and face recognition (FR) system architecture previously proposed for user-authentication applications [12,13]. For the FD operation, Figure 2a uses analog Haar-like filter circuit (AHFC) while Figure 2b uses analog CNN circuits. As shown in Figure 2b, the ADC is eliminated to optimize the power consumption, compared to [12]. However, both systems require the analog memory block to keep 20 or 3 rows to perform AHFC or analog CNN using the sub-windows. In other words, two phases of operation are required: the read-out operation of the CIS, using a rolling shutter; and the column-parallel read-out and sequential FD operation in the memory blocks. As a result, the total processing time is increased because of the memory blocks, leading to low-speed operation (~1 fps) for user-authentication applications in which real-time operation (frame rate ≥ 30) is necessary. Therefore, to obtain a power-efficient CIS with a high frame rate that can classify images in mobile devices, we propose a CIS integrated with always-on image classification, using an analog lightweight CNN (a-LWCNN) without the analog memory.

Figure 2c shows the proposed CIS integrated with an analog convolution processor in a chip. The FD in the proposed CIS is a binary classification, which assesses whether there is a face or not on the image frames. It should be noted that the proposed CIS supports FD-only, unlike previous works in [12,13] that support FD and FR. Since the proposed a-LWCNN circuits can be implemented in column-parallel circuits, which are correlated double sampling (CDS), pixel data of every row can be read out to perform convolution functions simultaneously, thereby improving processing speed and enabling real-time operation (with a maximum frame rate of 120 fps). Because the proposed CIS integrates the full process of the a-LWCNN, including an analog processing unit for convolution and pooling layers and a digital processing unit for the fully connected (FC) layer, we could obtain a high-area and power-efficient CIS. For the high-speed and low-power CIS integrated with a-LWCNN in a chip, the main contributions of this paper are as follows: 1) The CIS is optimized with the elimination of the ADC and memory blocks for low-power operation (1.46 μW of power consumption in the CNN). 2) The column-parallel CDS circuits support the operation of the analog CNN without analog memory that increases the frame rate (<120 fps). 3) The proposed column-parallel a-LWCNN circuits can operate without an operational transconductance amplifier (OTA), resulting in the reduction of static current in each column. The contents of the paper are as follows: Section 2 discusses the proposed CIS for image classification, including circuit design and implementation; Section 3 outlines the experimental results; and Section 4 provides a conclusion.

## 2. Design of the Proposed Functional CIS for Image Classification

### 2.1. The Proposed Image Classification with the a-LWCNN Algorithm

To integrate the CIS and LWCNN-based image classification in a single chip, we suggest using an a-LWCNN algorithm in the analog circuit domain. Conventionally, a CNN algorithm includes the convolution layer, pooling layer, and FC layer [14,15,16]. The main characteristics of the CNN structure mostly originate from the convolution and pooling operations. The convolution operations slide across the input values with filters consisting of learnable weights. The pooling operations reduce the dimension of the output values, which derive multiple values into one by extracting the average, minimum, and maximum value. In addition, the convolution operation has the key aspects of parameter sharing and sparse interaction between layers, and the pooling operation reduces the computational burden and the possibility of over-fitting. Since the algorithm allows the CNN to process large receptive fields with even fewer learnable weights, a CNN can operate much more efficiently than the typical artificial neural networks in processing high-dimensional data, such as images [17,18,19]. In that case, if the image resolution is 160 × 120, and 160 column-parallel high-bit (8–12 bits) ADCs are required, leading to 19,200 times the A/D conversion [20,21,22,23]. However, this is inefficient in terms of power consumption and chip area for the CIS. Therefore, in this study, we used an analog convolution processor for convolution and pooling layers before ADC processing for data compression, which reduced the number of ADCs and occurrences of A/D conversion.

**Figure 3:**
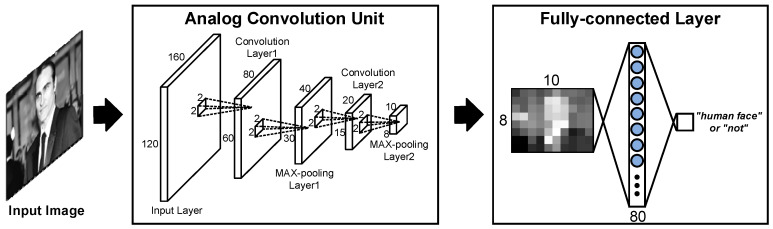
The proposed lightweight convolutional neural network (LWCNN) algorithm.

Figure 3 illustrates the proposed a-LWCNN algorithm. Unlike with the conventional implementation of a CNN algorithm, our network comprises four layers (1st convolution + 1st pooling + 2nd convolution + 2nd pooling) in the analog domain and an FC layer in the digital domain. Because all convolution and pooling layers use 2 × 2 filters and 2 × 2 strides, the data size can be reduced by one quarter for each layer, and the image size of 160 × 120 is reduced to 10 × 8 by passing all four layers. The 160 (# of columns) × 120 (# of rows) images become 80 × 60 after the 1st convolution processing. The convolution data are further reduced to a quarter, 40 × 30, since the max pooling circuit takes the maximum value of four neighboring pixels. After processing the 2nd layer as same as the 1st layer, the data size finally becomes 10 × 8. The 80 pieces of compressed data are finally converted to the digital domain through ADCs to determine the FC layer in the digital domain. It should be noted that the ADCs in the CIS consume the majority of the power (>50% [24]). Using the proposed CIS structure, the ADCs’ power consumption can be reduced from 160 to 10 (93.75% reduction), and the A/D conversion occurrence can be reduced from 19,200 to 80 (99.58% reduction) with data compression.

### 2.2. Overall Architecture of the Proposed CIS

**Figure 4:**
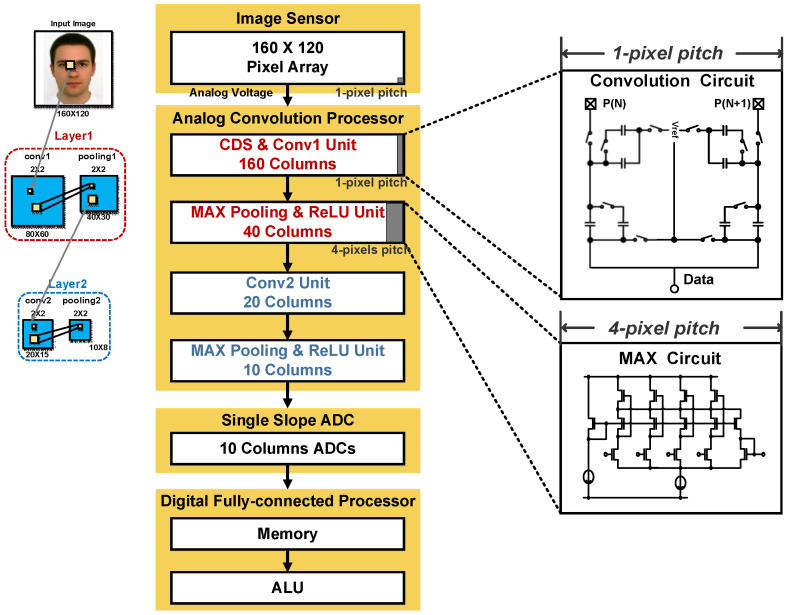
Overall architecture of the proposed Complementary Metal Oxide Semiconductor (CMOS) image sensor (CIS).

Figure 4 shows the overall architecture of the proposed CIS for image classification. The entire system is integrated with a CIS and an a-LWCNN-based image classification processor in a single chip. The architecture consists of a 160 × 120 pixel array, an analog convolution processor, 10 columns of single-slope ADCs, and a digital FC processor. The pixels are read out row by row using a rolling shutter. The analog convolution processor consists of a convolution circuit and a MAX circuit. The convolution circuit performs CDS and calculates the partial sum of the 2 × 2 weight filter. Then, the MAX circuit performs max pooling by searching for the maximum value. A rectified linear unit (ReLU) is operated by adding one input unit and applying a constant reference voltage. Data compressed on the analog domain through the analog convolution processor are converted to digital data using a 4-bit single-slope ADC. The digital FC processor consists of the memory and arithmetic logic unit (ALU). Data converted to digital code are stored in memory, and the ALU performs the FC layer consisting of 4-bit weights.

### 2.3. Detailed Building Blocks

**Figure 5:**
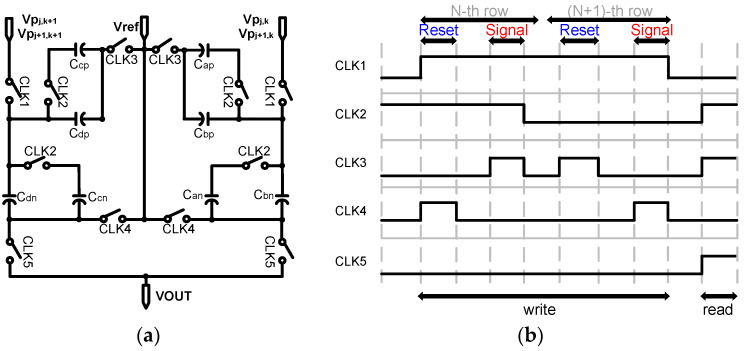
(**a**) Analog convolution circuit using a switched capacitor and (**b**) timing diagram.

**Figure 6:**
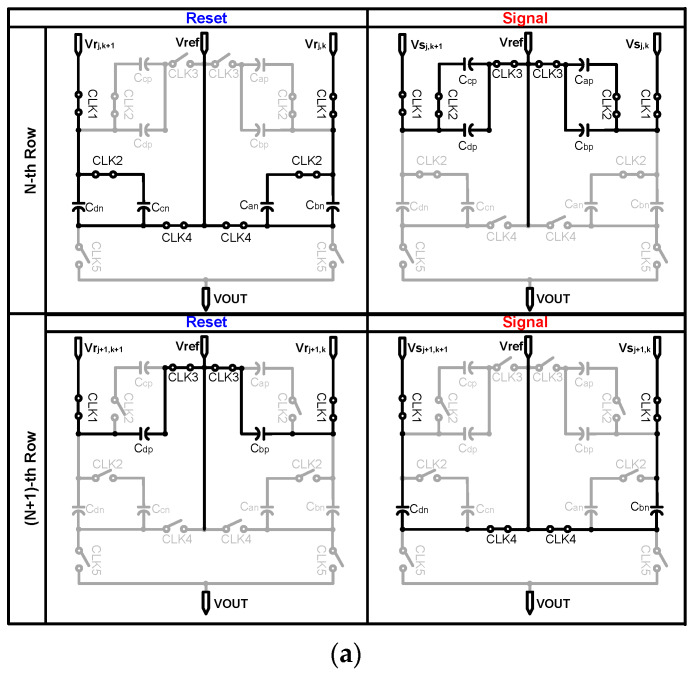

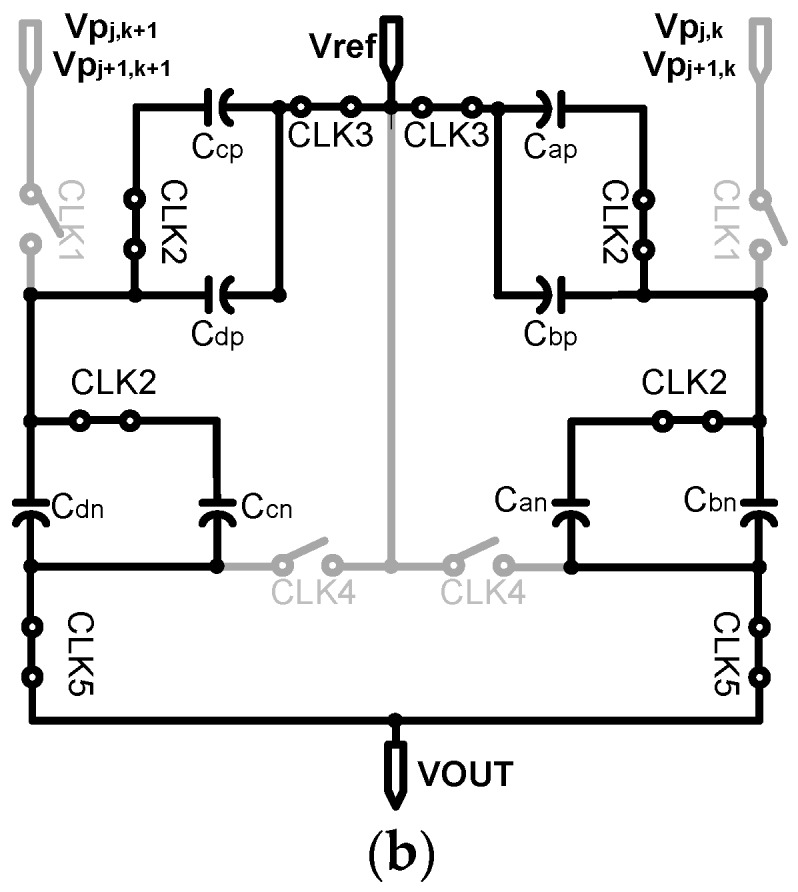
(**a**) The write operation of an analog convolution circuit, and (**b**) the read operation of an analog convolution circuit.

Figure 5a shows the structure of the proposed analog convolution circuit that serves as a convolution layer in this paper. To support the 2 × 2 weight filter operation, we utilized a switched-capacitor circuit without additional memory circuits. Furthermore, with 4T-APS, the convolution circuit also perform CDS to reduce noise from pixels [25] without an operational transconductance amplifier (OTA). For CDS, a pixel reset voltage, ${\mathrm{Vr}}_{j,k}$, is read out followed by the pixel signal value, ${\mathrm{Vs}}_{j,k}$, and then taking the difference (${\mathrm{Vp}}_{j,k}={\mathrm{Vr}}_{j,k}-{\mathrm{Vs}}_{j,k}$, where j is the row and k is the column). Therefore, we used two capacitors ($C_{p}{\;\mathrm{and}\;C}_{n}$) for ${\mathrm{Vr}}_{j,k}$ and ${\mathrm{Vs}}_{j,k}$, respectively, to store and subtract the pixel values. Figure 5b shows the timing diagram of the CDS and CNN operations. Because the proposed CIS uses a rolling shutter operation, the pixels are read out row by row, and each row has a reset and signal phase for CDS; the timing diagram consists of write and read phases as shown below. Figure 6a,b shows the operation of the analog convolution circuit in the write phase and read phase, respectively.

1. First, ${\mathrm{Vr}}_{j,k}$ is sampled onto $C_{\mathrm{an}}$ and $C_{\mathrm{bn}}$. As the output of the pixel changes from reset to signal, ${\mathrm{Vs}}_{j,k}$ is stored in $C_{\mathrm{ap}}$ and $C_{\mathrm{bp}}$;
2. By the row scanner, the pixel is changed from the nth row to the (n + 1)th row; ${\mathrm{Vr}}_{j+1,k}$ is sampled only onto $C_{\mathrm{bp}}$ as the switch that is used to connect $C_{\mathrm{ap}}$, and $C_{\mathrm{bp}}$ is opened by CLK2. Next, ${\mathrm{Vs}}_{j+1,k}$ is sampled onto $C_{\mathrm{bn}}$, and each of the four capacitors stores a different value. Similarly, the (k + 1)-column also performs this operation to store the values for ${\mathrm{Vp}}_{j,k+1}$ and ${\mathrm{Vp}}_{j+1,k+1}$ in $C_{\mathrm{cp}}$, $C_{\mathrm{dp}}$, $C_{\mathrm{cn}}$, and $C_{\mathrm{dn}}$;
3. In the read phase, reference voltage is applied in one direction to average the four pixel values stored in each of the capacitors. The final voltage at the output of the convolution circuit is ideally as given by Equation (1):
  (1) $$V_{\mathrm{OUT}}=V_{\mathrm{REF}}-\left(\frac{{\mathrm{Vp}}_{j,k}\times C_{a}+{\mathrm{Vp}}_{j,k+1}\times C_{b}-{\mathrm{Vp}}_{j+1,k}\times C_{c}-{\mathrm{Vp}}_{j+1,k+1}\times C_{d}}{C_{a}+C_{b}+C_{c}+C_{d}}\right)$$

For example, if the (−2, −1, 1, 2) filter is used, the capacitances are $C_{a}$ = 1 pF, $C_{b}$ = 0.5 pF, $C_{c}$ = 0.5 pF, and $C_{d}$ = 1 pF. When $V_{\mathrm{REF}}$ = 2 V, ${\mathrm{Vp}}_{j,k}$ = 0.2 V, ${\mathrm{Vp}}_{j,k+1}$ = 0.5 V, ${\mathrm{Vp}}_{j+1,k}$ = 0.4 V, and ${\mathrm{Vp}}_{j+1,k+1}$ = 0.1 V, the final voltage,${\;V}_{\mathrm{OUT}}$, becomes $2\;V-\frac{0.2\;V\times 1\;\mathrm{pF}+0.5\;V\times 0.5\;\mathrm{pF}-0.4\;V\times 0.5\;\mathrm{pF}-0.1\;V\times 1\;\mathrm{pF}}{1\mathrm{pF}+0.5\mathrm{pF}+0.5\mathrm{pF}+1\mathrm{pF}}=1.925\;V$. It should be noted that in the analog convolution circuits, transmission gates are used for all switches to cover input voltage range and to minimize charge injection. All capacitors are MIM capacitors, and unit capacitance is 0.5 pF in this paper.

**Figure 7:**
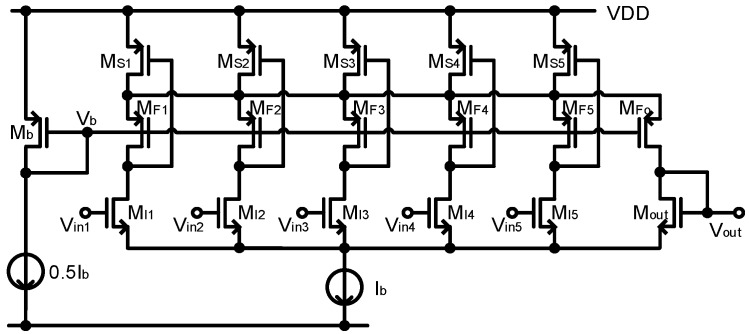
The voltage-mode MAX circuit.

**Figure 8:**
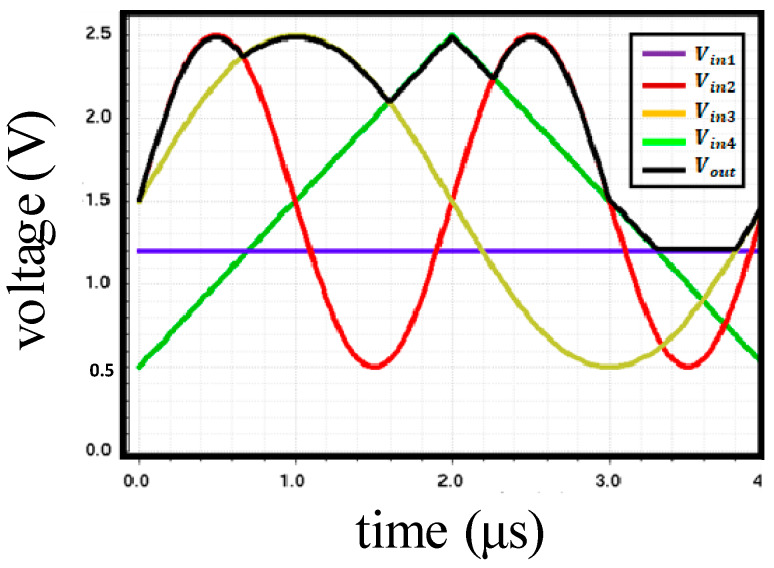
Simulation results of the voltage-mode MAX circuit.

For the max pooling, we used a voltage-mode MAX circuit that is widely used in neural networks [26], called a max-pooling processor. In this circuit, output voltage is equal to the maximum input voltage. Figure 7 shows the structure of the voltage-mode MAX circuit, which contains an nMOS of common-source strategy and a current mode pMOS section. Each unit is composed of three transistors: an input transistor ($M_{\mathrm{Ii}}$) that is connected to other input devices at the source node; a cascode transistor ($M_{\mathrm{Fi}}$) that is biased with a fixed voltage; and a current source transistor ($M_{\mathrm{Si}}$), which is connected to other similar features at a drain node. The corresponding device of the maximum voltage unit operates in a saturation region, and other devices enter either the triode or cutoff regions. Therefore, the current flow and the maximum voltage can be copied to $M_{\mathrm{Fo}}$ with a current mirror. Figure 8 shows the simulation results of the MAX circuit for four different-input cases. Although the waveforms of V_in_ in the MAX circuit are different, such as DC, pulse, sine, and triangle waveform, V_out_ tracks the maximum voltage (see the black line in Figure 8). Since our system uses a 2 × 2 filter, max pooling is possible through four inputs. However, by adding one unit and applying a constant reference voltage, the output voltage always maintains a higher value than the reference voltage to perform the ReLU operation.

**Figure 9:**
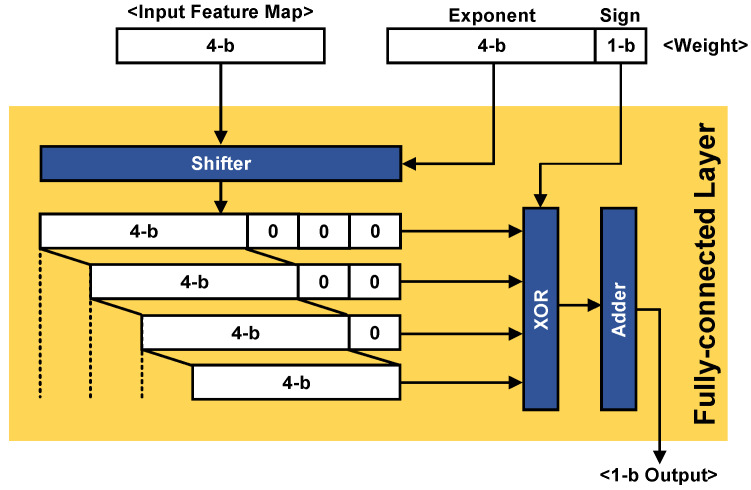
Block diagram of the fully connected layer.

After analog convolution processing in the analog domain, data were reduced from 160 to 10 columns. As mentioned earlier, image data size (160 × 120) can be reduced by one quarter for each layer, finally resulting in 10 × 8 (two convolution units and two pooling units with a 2 × 2 filter and stride, as shown in Figure 4). The column data were converted to the digital domain with the 4-bit single-slope ADC. For the FC operation, a 4-bit fixed-point number and 5-bit floating-point number were used for the feature maps and weights, respectively. Figure 9 shows the block diagram of the proposed FC unit. With the weights represented by the floating point, which is composed of a 1-bit sign and a 4-bit exponent, the FC unit is implemented by using a shifter, an exclusive or (XOR), and an adder instead of a multiplier. After all processes are complete, the processor outputs a single-bit result that can classify whether there is a face or not in the images.

## 3. Experimental Results

### 3.1. Chip Measurement Results

**Figure 10:**
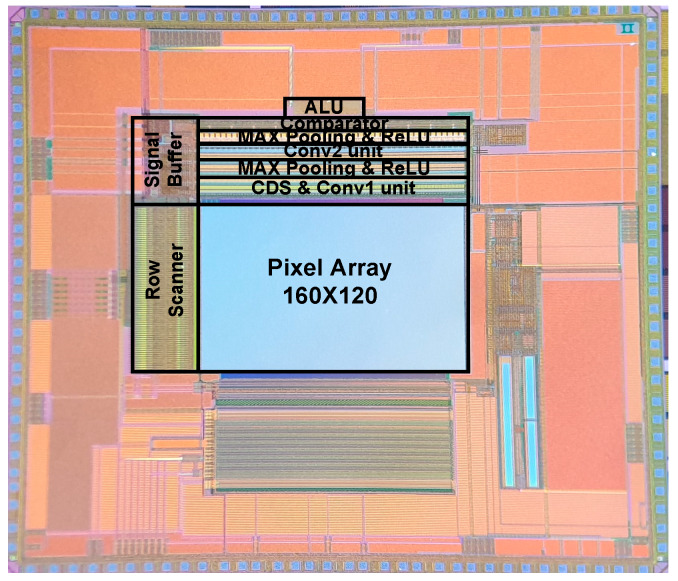
Chip photograph of the proposed CIS.

The proposed CIS has a Quarter Quarter Video Graphics Array (QQVGA) resolution (160 pixel × 120 pixel array) with a 3.3 V supply voltage and a 0.11 μm 1-poly 4-metal CIS process. Figure 10 shows the chip photograph of the proposed CIS integrated with always-on image classification using an a-LWCNN. The total area of the chip, including the I/O pads, is 5.90 mm × 5.24 mm, and the effective area is 7.65 mm^2^. The total power consumption is 0.96 mW with a 3.3 V supply voltage at 60 frames/s. Table 1 gives the detailed specifications of the proposed CIS.

**Figure 11:**
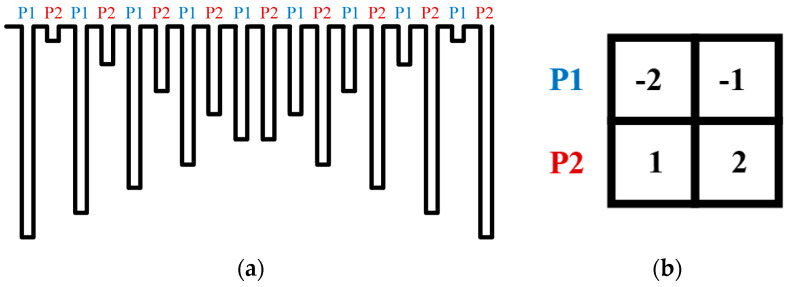
(**a**) Digital-to-analog converter (DAC) signal for the test mode. (**b**) Weight values of the 1st layer of the CNN.

**Figure 12:**
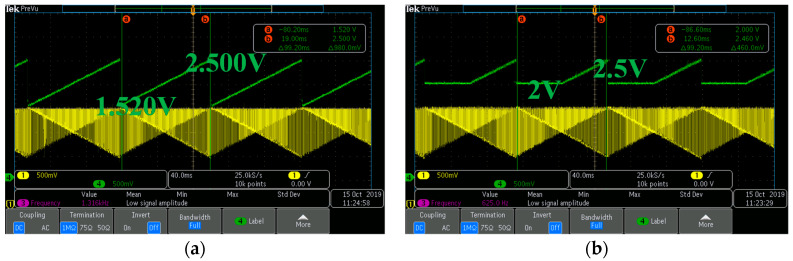
Measurement results in (**a**) the convolution circuit and (**b**) the MAX circuit.

We measured the chip using a Field-Programmable Gate Array (FPGA) board to generate the control signals required for a convolution circuit, ALU blocks, and other operations; the signal generated by Xilinx was applied to the design circuit through the motherboard using the FPGA board. The chip includes a test mode that allowed us to measure the analog processor by accessing the inputs of the convolution circuits. We controlled the external digital-to-analog converter (DAC) with signals from the FPGA to artificially generate a reset and signal voltage of 4T-APS, and we measured the performance of the circuit by applying it to the test bench. Figure 11a shows the output of the DAC that is accredited to the inputs of the convolution circuits, and Figure 11b shows the weights of the filter used for image classification. In the first row, P1, the multiplied weights have negative values, and the reset voltage and the signal voltage difference, the pixel value, decreases from maximum to minimum. Ideally, therefore, the output of the convolution circuit increases with the slope from minimum (1.5 V) to maximum (2.5 V). Then, by applying a constant reference voltage (2 V) to the MAX circuit, we observed that the ReLU operation is correct. The oscilloscope measurement results are plotted for both the convolution (Figure 12a) and the MAX (Figure 12b) circuits.

### 3.2. Classification Results

We implemented the a-LWCNN model for the image classification system with 4513 grayscale images that were 160 × 120. The image dataset consisted of 1666 positive images, frontal images of human faces, and 2847 negative images. We split the dataset into training data, validation data, and test data. The weights of the a-LWCNN model were trained using a back propagation algorithm [27] with training data, and we employed an early stopping algorithm [28] using validation data to prevent over-fitting. We used four performance measures, namely accuracy, precision, recall, and specificity, which are the most commonly used for image classification. The different performance measures can be described as follows: (1) accuracy: the ratio of the number of instances correctly classified to the total number of instances; (2) precision: the ratio of the number of positive instances correctly classified to the number of instances predicted as positive; (3) recall: the ratio of the number of positive instances correctly classified to the number of positive instances; and (4) specificity: the ratio of the number of negative instances correctly classified to the number of negative instances. The importance of these performance measures may vary depending on preference, and users can reflect their preferences by adjusting the bias value of the last fully connected layer of the a-LWCNN. The bias controls the overall tendency of the classification: a higher bias tends to classify an input image into the positive class, meaning that a face exists, and lower bias leads the classifier to perform in the opposite way. Thus, users who want to achieve high precision rather than other performance measures can decrease the bias, and those who need to focus on accuracy should find an appropriate bias to maximize the accuracy [29,30,31]. Typically, this tendency of classification can be determined through the cut-off value. However, in this study, it was controlled through the bias for the implementation of the circuit. The bias of the last fully connected layer moves the output value of the a-LWCNN in parallel, so it plays the same role as controlling the cut-off value.

**Figure 13:**
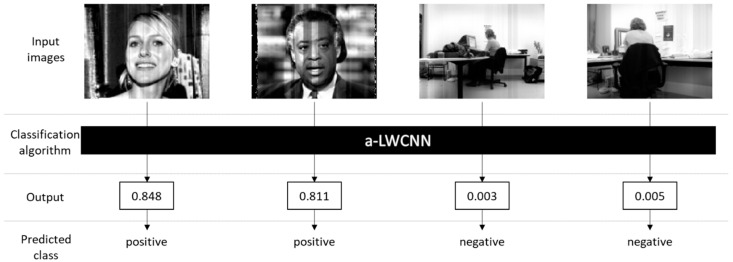
Examples of the face recognition process.

In order to design a convolution circuit, we used the unit capacitor to reduce mismatch, and to implement this, we quantized the weights obtained by training; the accuracy loss was less than 0.5%. Examples of the face detection process using an a-LWCNN are shown in Figure 13. The left two input images are from the positive class, and the right two images belong to the negative class. The a-LWCNN processes the input images and then outputs values between 0 and 1: the predictive probabilities that the inputs belong to the positive class. The two left images are classified as positive with output values of 0.848 and 0.811, and the others are classified into the negative class since their output values are 0.003 and 0.005, providing the cut-off value is set to 0.5. The time needed for the proposed face detection is 5.207 × 10^−05^ s per image on the computer we used under the following specifications: Processor: Intel(R) Core(TM) i7-7700K CPU; RAM: 64.0 GB; OS: Windows 10; and we also used Deep Learning Library PyTorch 1.3.1.

**Figure 14:**
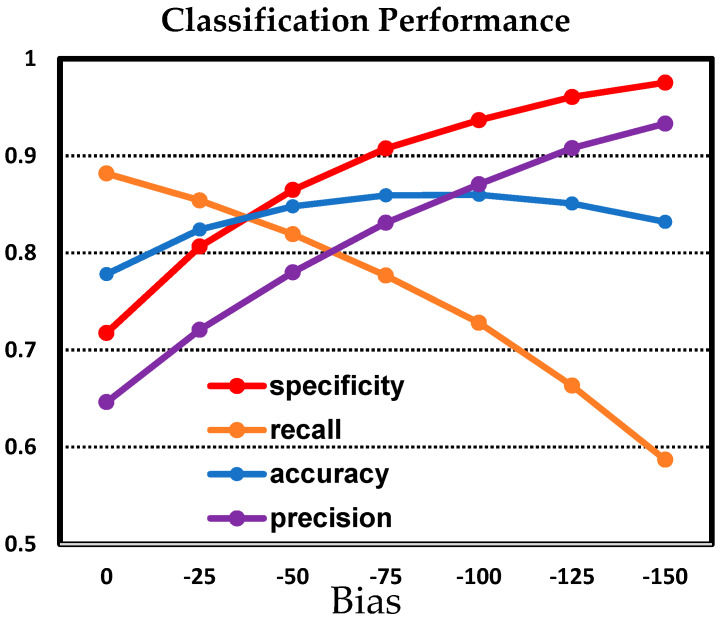
Performance of the proposed CIS.

Table 2 shows the confusion matrix of the proposed method with the highest accuracy. The confusion matrix shows the performance of a classification algorithm. Each row and column of the confusion matrix represent the images in an actual class and predicted class, respectively. The a-LWCNN correctly classified 72 positive images and 196 negative images of test data. On the other hand, 28 positive images and 4 negative images were misclassified. Therefore, 268 of the 300-image test data were correctly classified, making the accuracy was 89.33%. Figure 14 shows the accuracy, recall, specificity, and precision according to the bias. When the bias was −64, the accuracy was 89% and the precision was 90%, and when the bias was −128, the accuracy was 85% and the precision was 95%. In other words, when the bias can be controlled to be low enough, accuracy and precision have a trade-off relationship.

### 3.3. Discussion

Table 3 shows the performance comparison with state-of-the-art works. Unlike the previous works in [12,13] that support both face detection (FD) and face recognition (FR), the proposed CIS supports FD only. Since the FR system in [12,13] requires an additional CNN processor, we show the comparison results of the FD-related performances. The power consumption of convolution for FD (1.46 μW) of the analog convolution circuits in the proposed CIS is about 10 times lower than that of [13]. Because the proposed a-LWCNN circuits can perform CDS and convolution functions without an OTA, the static power consumption in the column parallel circuits is quite low. In addition, as mentioned earlier, the a-LWCNN circuit can be implemented in a column-parallel structure without using analog memory blocks. Therefore, we could obtain the maximum frame rate of 120 fps, while those of others are 1 fps. In terms of total power consumption, the proposed CIS shows a slightly higher power consumption compared to [12,13], since the designed MAX circuit consumes the high static current, resulting in 90% of the total power consumption (about 1 mW at 120 fps). Therefore, using a low power MAX circuit like the dynamic MAX circuit, the total power consumption can be further reduced. This study obtained the fastest processing speed of up to 120 fps while maintaining low power consumption, 1.12 mW. Furthermore, the power consumption is a function of the frame rate, which is about 15–44% of the power reduction with a 10× decrease in the frame rate [32,33]. With the low frame rate, the estimated total power consumption of the proposed CIS is 0.16 mW with 1 fps. Because the application of the proposed always-on CIS is a trigger to turn on power-hungry mobile devices when there is a face, an 89.33% accuracy with a high frame rate is acceptable, while FR requires a high accuracy for accurate user identification.

In terms of the actual use of the a-LWCNN as an always-on device, it is possible to waste power if it is used as a trigger to turn on another high-power device. In actual use, there are many more cases where a face does not exist than cases where a face exists. Therefore, a high probability of classifying an image as positive can prevent a waste of power, and it can be achieved by increasing the precision. In addition, the frame rate of the image sensor we used is normally 60 fps and 120 fps at maximum, so the a-LWCNN can be used without inconvenience in practice under high precision. In this situation, it is reasonable to set the bias to a large negative value to obtain high precision.

## 4. Conclusions

In this paper, we propose an always-on CIS based on an analog LWCNN for image classification. Using the proposed CIS, images can be classified without a high-resolution ADC or additional memory blocks for CNN processing. We propose using an analog convolution circuit with the switched capacitor to compute the CNN convolution layer and to operate the CDS without an OTA under a low power budget (<1 uW per column). The proposed max-pooling processor with a voltage-mode MAX circuit served well in obtaining the maximum multi-input voltages for the ReLU function. Since the static power consumption of the MAX circuit is dominant in total power consumption, by replacing the static MAX circuit with a dynamic MAX circuit, power consumption could be dramatically reduced. With a lower stride, from 2 (in this paper) to 1, the accuracy could be improved. We believe that the proposed CIS can be used for ultra-low power image classification applications in mobile devices with limits to their power consumption.

## Figures and Tables

**Table 1:** Performance summary of the proposed CIS.

Process Tech.	0.11 μm 1P4M CIS Process
Chip Size	5.90 mm × 5.24 mm(30.92 mm^2^)
Core Size	2.93 mm × 2.61 mm(7.65 mm^2^)
Resolution	QQVGA (160 × 120)
Pixel type	4T-APS
Supply voltages	3.3 V (Analog)/1.5 (Digital)
Power consumption	0.96 mW @ 60 fps/1.12 mW @ 120 fps
Maximum Frame rate	120 fps

**Table 2:** Confusion matrix of the proposed model (*n* = 300).

Actual	Predicted	Positive	Negative
**Positive**		72	28
**Negative**		4	196

**Table 3:** Performance comparison.

	JSSC’18 [12]	ISCAS’19 [13]	This Work
**Technology**	Samsung 65 nm	Samsung 65 nm	Dongbu 110 nm
**Algorithm**	FD: Haar-like FR: Digital CNN	FD and FR: Analog–Digital Hybrid CNN	FD: Analog-CNN
**Accuracy**	97%	96.18%	89.33%
**Resolution**	QVGA	QVGA	QQVGA
**Conv. Power**	24–96 μW ^1^	10.17–18.75 μW ^2^	1.46 μW ^2^
**Total Power**	0.62 mW @ 1 fps ^3^	0.62 mW @ 1 fps ^3^	0.16 mW @ 1 fps ^4^
			0.96 mW @ 60 fps
			1.12 mW @ 120 fps

^1^ Power consumption using an analog Haar-like algorithm for FD [12]. ^2^ Power consumption using an analog CNN for FD [12]. ^3^ Total power consumption of FD and FR (in CNN processor). ^4^ Estimated power consumption for FD (15% reduction/10 fps); power consumption is reduced by about 15–44% with the reduction of every 10 fps in [32,33].
